# High neutrophil percentage and neutrophil-lymphocyte ratio in acute phase of ischemic stroke predict cognitive impairment: A single-center retrospective study in China

**DOI:** 10.3389/fneur.2022.907486

**Published:** 2022-08-22

**Authors:** Tianling Shang, Bo Ma, Yanxin Shen, Chunxiao Wei, Zicheng Wang, Weijie Zhai, Mingxi Li, Yongchun Wang, Li Sun

**Affiliations:** ^1^Department of Neurology, The First Hospital of Jilin University, Changchun, China; ^2^Department of Hepatology, The First Hospital of Jilin University, Changchun, China

**Keywords:** post-stroke cognitive impairment, acute ischemic stroke, inflammation indicators, neutrophil percentage, neutrophil/lymphocyte ratio

## Abstract

**Background and aims:**

Recently, various hemocyte and blood cell ratios have garnered researchers' attention, as a low-cost, widely prevalent, and easy-to-measure index for diagnosing and predicting disease. Therefore, we sought to investigate the effect and predictive value of the peripheral blood neutrophil percentage and neutrophil-lymphocyte ratio (NLR) in the acute phase of ischemic stroke (AIS) in post-stroke cognitive impairment (PSCI).

**Methods:**

We selected 454 patients with mild AIS and acquired general clinical data. The patients were divided into PSCI and post-stroke no cognitive impairment (PSNCI) groups according to their Montreal Cognitive Assessment (MOCA) scores. We assessed whether there were differences in clinical data, peripheral blood neutrophil percentage, and NLR values between the different groups. We also analyzed the independent influences on the occurrence of PSCI using a binary logistic regression. Receiver operating characteristic (ROC) curves were used to analyze the predictive value of the above inflammatory indicators and models containing different inflammatory indicators for PSCI.

**Results:**

In total, 454 patients were included, of whom 253 (55.7%) patients were in the PSCI group, with a mean age of 62.15 ± 7.34 years and median neutrophil percentage and NLR of 0.64 (0.32–0.95) and 2.39 (0.71–54.46), respectively. Both neutrophil percentage (adjusted OR = 1.025; 95% confidence interval: 1.005–1.406) and NLR as a categorical variable (Q5, adjusted OR = 2.167; 95% CI: 1.127–4.166) were independent risk factors for PSCI, and the Q5 group (NLR ≥ 4.05) had significantly worse overall cognition and executive function.

**Conclusions:**

Neutrophil percentage and NLR in the acute phase of AIS were independently associated with PSCI, and a high NLR was strongly associated with executive function. In addition, neutrophil percentage and NLR have diagnostic values for PSCI.

## Introduction

Post-stroke cognitive impairment (PSCI) is a clinical syndrome characterized by cognitive impairment beginning post-stroke and lasting until 6 months. It occurs in approximately one-third of stroke patients (1). Although PSCI is quite prevalent, it does not receive sufficient attention in the acute or early stages of stroke and often goes unnoticed by patients and their families until significant memory loss, slowed reaction time, and appearance of psychiatric symptoms following failure to seek prompt cognitive treatment. Therefore, it is particularly important to identify and screen for patients with PSCI at an early stage in clinical practice. Recently, studies have found that peripheral blood neutrophil count and NLR are linked to cognitive impairment (CI) and are low cost and easy to obtain. However, the association between these factors and PSCI remains to be elucidated.

The exact pathophysiological mechanisms underpinning PSCI remain unclear. It is currently believed that the triad of hypoperfusion, oxidative stress, and inflammation, acting alone or in combination, affects the neurovascular unit and is thus involved in the pathogenesis of vascular cognitive impairment. Inflammation and immune response are involved in the pathogenesis of AIS (2, 3). Prior to disease onset, abnormal immune responses can induce inflammation within and around the vessel wall, which promotes thrombosis, alters vascular reactivity, and promotes atherosclerosis. Within hours of the onset of ischemic stroke, peripheral blood neutrophil counts increase exponentially (4) and lymphocyte counts decrease exponentially. Neutrophils exert a destructive effect on the blood-brain barrier (BBB) by producing reactive oxygen species (ROS) and synthesizing cytokines, chemokines, and intercellular adhesion molecules (5), and they degranulate to produce myeloperoxidase (MPO) and neutrophil elastase (NE) (6), which in turn may exacerbate damage to the neurovascular unit through a vicious cycle between inflammation and BBB destruction (7). Numerous studies have demonstrated that damage to the neurovascular unit is associated with the onset of cognitive impairment (8). We sought to further understand the pathogenesis of PSCI by exploring the association between the percentage of neutrophils, NLR, and PSCI in the acute phase of AIS to better screen and treat PSCI.

## Materials and methods

### Study population

**Figure 1:**
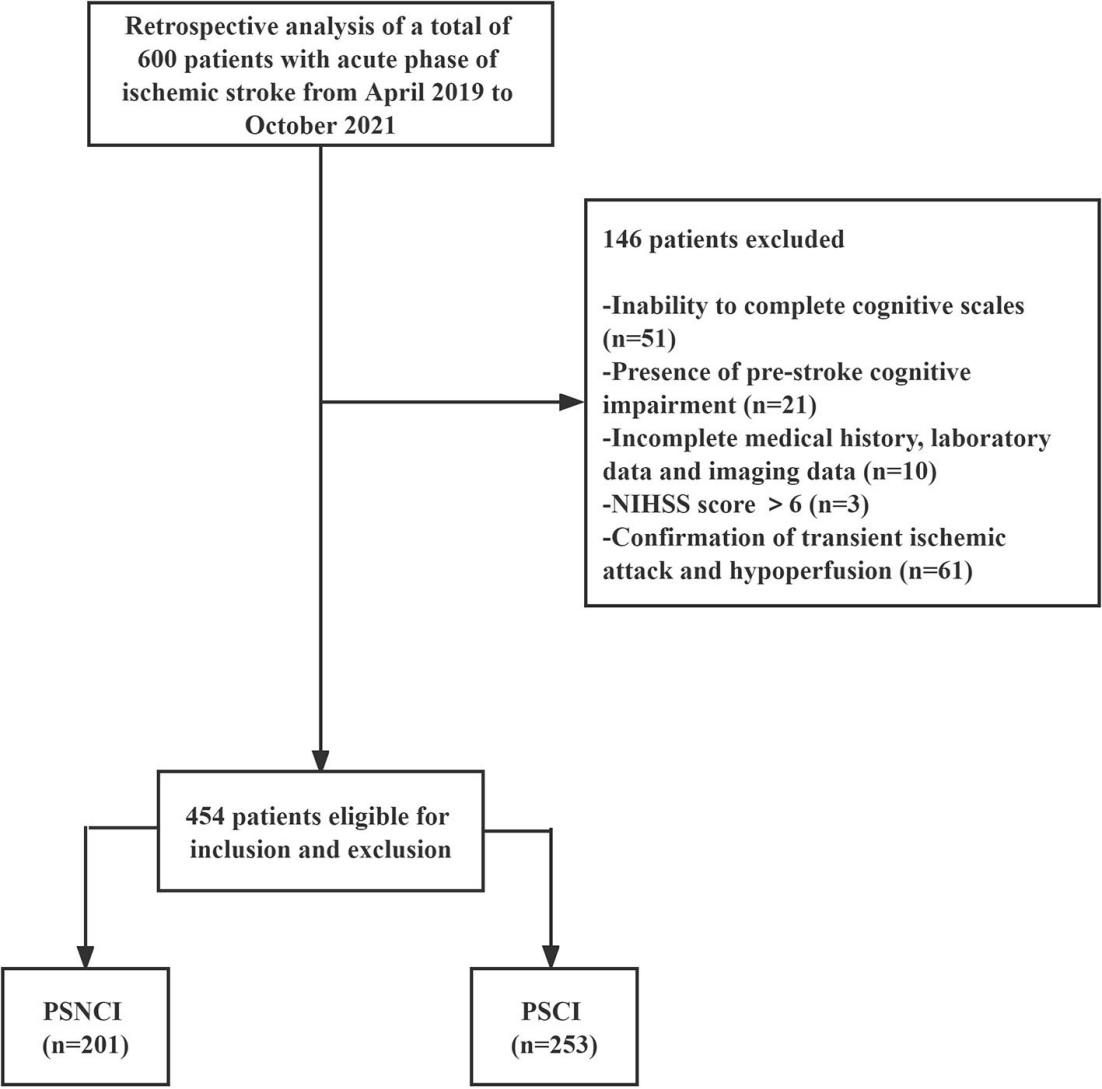
Study flow diagram. PSCI, post-stroke cognitive impairment; PSNCI, post-stroke no cognitive impairment; NIHSS, National Institute of Health Stroke Scale.

In total, 454 patients with mild AIS who had been admitted to the Department of Neurology at the First Hospital of Jilin University between April 2019 and October 2021 were selected. The flow diagram of the study is demonstrated in Figure 1. The inclusion criteria were as follows: (i) met the WHO diagnostic criteria for acute ischemic stroke (9) and were aged 50–80 years; (ii) had symptoms of focal neurological deficit and new infarcted lesions on brain magnetic resonance (MR) diffusion-weighted imaging (DWI); (iii) National Institutes of Health Stroke Scale (NIHSS) score ≤ 6; (iv) were informed and signed informed consent; and (v) were able to cooperate in completing relevant tests and cognitive scales. The exclusion criteria were as follows: (i) cognitive impairment due to a combination of medical or surgical systemic disease, infectious disease, toxic metabolic disease, or psychiatric disease; (ii) history of memory loss, diagnosis of cognitive impairment prior to stroke, or use of medication affecting cognition in the prior 2 weeks; and (iii) inability to complete cognitive scales due to visual impairment, aphasia, deafness, or severe limb immobility; (iv) patients with various infectious, neoplastic, hematological, or other diseases that have caused alterations in their peripheral blood picture in the last 2 weeks or the corresponding treatment. The study protocol was approved by the Ethics Committee of the First Hospital of Jilin University and was conducted according to the principles of the Declaration of Helsinki. Written informed consent was obtained from all participants.

### Study variables

We acquired general clinical data during hospitalization, including age, sex, education level, body mass index (BMI), and systolic and diastolic blood pressure measured for the first time after admission. The internationally accepted trial of org 10172 in acute stroke treatment (TOAST) typing was used to classify cerebral infarction according to its cause, including large artery atherosclerosis (LAA), cardiogenic embolism (CE), small artery occlusion (SAA), other causes (OD) and unexplained (UD) (10). NIHSS was used to assess the severity of neurological deficits (11) and scaled from 0 to 6.

Routine blood samples were acquired within 24 h of AIS occurrence to examine for neutrophil percentage, absolute neutrophil value, and absolute lymphocyte value. Fasting blood glucose, various lipids, uric acid, cystatin C, homocysteine, and CRP were collected on the following day, while NLR was equal to the absolute neutrophil value/absolute lymphocyte value. The study subjects were placed into five groups according to the NLR quintiles: Q1 (≤ 1.62), Q2 (1.63–2.08), Q3 (2.09–2.72), Q4 (2.73–4.04), and Q5 (≥4.05). All blood samples were obtained from the Biobank of the First Hospital of Jilin University. Complete blood count was measured using a SYSMEX XN-9000 hematology analyzer (Sysmex Corporation, Kobe, Japan) according to the manufacturer's recommendation.

The following imaging measures were collected within 72 h of AIS: the diameter of maximum transverse section (DMTS) of the infarct was measured based on brain MRI DWI axial position to assist in the assessment of infarct size in AIS. For the convenience of the analysis, the brain circulation was divided into anterior circulation, including frontal, parietal, lateral temporal cortex and subcortical areas, internal capsule and basal ganglia, and posterior circulation, including brainstem, cerebellum, thalamus, medial temporal lobe, and occipital areas. In addition, to analyze whether the infarct number affected PSCI, we included either a single or two numbers. We also analyzed the effects of white matter loose grade on PSCI, including periventricular hyperintensity (PVH) and deep white matter hyperintensity (DWMH) through imaging. All of the imaging data described above were evaluated by a professional imaging physician.

On days 7 to 10 following AIS, when the symptoms of cerebral infarction were relatively stable, cognitive psychological assessments were carried out on all included patients by psychometrically qualified assessors in a relatively quiet environment, without any other patient present at the same time. The overall cognitive function was assessed using the MOCA, with a total score of 30. MOCA is strongly correlated with educational level; studies have revealed that the corrected MOCA score increased by 1 when the length of education is <12 years (12). We classified the study subjects into PSCI (MOCA < 22) and PSNCI (MOCA ≥ 22) groups based on the adjusted MOCA (13). Assessment of various cognitive domains, including memory, language, attention, visual space, and execution ability, was performed using the auditory word-learning test-long delayed recall, verbal fluency test, connectedness test B, and MOCA-visual spatial and executive ability.

### Statistical analysis

Parametric continuous variables (e.g., BMI) are expressed as the mean (± standard deviation). Groups were compared using an independent Student's *t*-test, whereas nonparametric continuous variables (e.g., age, years of education, systolic pressure, diastolic pressure, DMTS, NIHSS, PVH, DWMH, various blood lipids, fasting blood glucose, cystatin C, uric acid, homocysteine, CRP, leukocytes, neutral ratio, lymphatic ratio, neutral value, lymphatic value, and NLR) are represented as the median (quartile 25-quartile 75). Groups were compared using a Mann–Whitney U test. Categorical variables (e.g., sex, previous medical history, TOAST, infarct site and number, and NLR quintile) are presented as the number (*n*) and proportion (%). Groups were compared using a chi-squared test. Univariate and multivariate logistic regression analyses were used to investigate the correlation between percent neutrophils, NLR, and PSCI. Multivariable models were adjusted for variables with a *p* < 0.1 in the univariate analysis. Univariate included general clinical data (e.g., age, sex, education level, BMI, blood pressure, NIHSS, and TOAST), various laboratory indicators, cognitive scores, and imaging data (e.g., DMTS, PVH, and DWMH). An association was indicated as the odds ratio or adjusted odds ratio (aOR) with the 95% confidence interval (CI). To evaluate the collinearity among the different peripheral blood inflammation indicators, a regression analysis was carried out to judge the independent influencing factors of PSCI. The ROC curve was used to analyze the predictive value of models containing different inflammatory indicators and single inflammatory indicators for PSCI. All statistical analyses were performed in SPSS version 23 (IBM, New York, NY, USA). Statistical significance was defined as a two-sided *P*-value of < 0.05.

## Results

### Characterization of patients with mild AIS

A total of 454 patients with mild AIS in the acute phase were included, of whom 253 (55.7%) patients were in the PSCI group, with a mean age of 62.15 ± 7.34 years and median neutrophil percentage and NLR of 0.64 (0.32–0.95) and 2.39 (0.71–54.46), respectively. There were 326 males (71.8%), and their median level of education was 9 (0–19) years. According to TOAST typing, the LAA had the largest proportion of cerebral infarcts (52.2%), followed by the SVO (37.7%). Only 25.8% of patients had infarcts in key memory-related sites such as the thalamus, hippocampus, medial temporal lobe, and angular gyrus; the median DWMH and PVH grades were 2 and 1, respectively. As regards past medical history, the prevalence rates of a positive history of hypertension, diabetes, previous stroke, heart disease, smoking, and drinking were 63.9, 31.1, 26.4, 17, 50.9, and 45.2%, respectively.

### Comparison between the PSCI and PSNCI groups in the acute phase of AIS

The PSCI group had a higher proportion of previous hypertension (68.0%) and stroke history (30.8%) than those of the PSNCI group (58.7%, 20.9%). PSCI occurred more frequently in patients with ≥ 2 infarct foci (50.2%) than in those with single infarct foci (38.3%), with the highest proportion of PSCI occurring in patients with anterior circulation infarction (53.4%), followed by those with posterior circulation infarction (35.2%), and both anterior and posterior circulation (11.5%). Patients in the PSCI group were older, had lower education levels, higher admission systolic blood pressure and overall NIHSS scores, larger DMTS and lower uric acid levels, and more severe DWMH. In addition, the two groups had statistically significant differences in memory, language, and executive and visuospatial areas, with the PSCI group having lower scores across all cognitive domains than that of the PSNCI group (*P* < 0.05) (see Table 1).

**Table 1:** Comparison of baseline characteristics between the PSCI and PSNCI groups.

	**PSNCI**	**PSCI**	***P-*value**
	**(*N* = 201)**	**(*N* = 253)**	
Sex-Female (*N*, %)	48 (23.9)	80 (31.6)	0.069
Hypertension (*N*, %)	118 (58.7)	172 (68.0)	0.041
Diabetes (*N*, %)	59 (29.4)	82 (32.4)	0.484
Heart disease (*N*, %)	35 (17.4)	42 (16.6)	0.819
History of stroke (*N*, %)	42 (20.9)	78 (30.8)	0.017
Smoking history (*N*, %)	108 (53.7)	123 (48.6)	0.279
Drinking history (*N*, %)	94 (46.8)	111 (43.9)	0.538
**Stroke subtype (TOAST)**			
LAA (*N*, %)	91 (45.3)	146 (57.7)	
CE (*N*, %)	3 (1.5)	2 (0.8)	0.085
SVO (*N*, %)	89 (44.3)	82 (32.4)	
OD (*N*, %)	3 (1.5)	5 (2.0)	
UD (*N*, %)	15 (7.5)	18 (7.1)	
Critical site infarction (*N*, %)	49 (24.4)	68 (26.9)	0.545
Number of infarcts (≥ 2) (*N*, %)	77 (38.3)	127 (50.2)	0.024
**Anterior/posterior circulation infarction**			
Anterior (*N*, %)	90 (44.8)	135 (53.4)	0.041
Posterior (*N*, %)	94 (46.8)	89 (35.2)	
Both (*N*, %)	17 (8.5)	29 (11.5)	
**On/off-screen infarction**			
On-screen (*N*, %)	126 (62.7)	164 (64.8)	0.122
Off-screen (*N*, %)	65 (32.3)	66 (26.1)	
Both (*N*, %)	10 (5.0)	23 (9.1)	
Age (years)	61 (54.5–67.5)	62 (58–69)	0.002
Education (years)	12 (9–15)	9 (6–12)	<0.001
Systolic pressure (mmHg)	145 (133–162)	150 (137–166)	0.030
Diastolic pressure (mmHg)	87 (78–94)	86 (78–96)	0.581
NIHSS (score)	2 (1–3)	2 (1–3)	0.008
DMTS (cm)	11.9 (7.48–14.1)	13.5 (8.915–17.755)	0.001
PVH	1 (1–2)	1 (1–2)	0.219
DWMH	1 (0.5–1)	1 (1–1.5)	0.017
Serum cystatin C (mg/l)	0.95 (0.86–1.07)	0.97 (0.87–1.105)	0.488
Fasting blood glucose (mmol/L)	5.58 (5.03–6.845)	5.74 (5.085–7.25)	0.473
Triglycerides (mmol/L)	1.55 (1.125–2.15)	1.58 (1.17–2.025)	0.985
Total cholesterol (mmol/L)	4.5 (3.835–5.145)	4.49 (3.82–5.21)	0.980
HDL-C (mmol/L)	0.98 (0.88–1.17)	1.01 (0.9–1.145)	0.570
LDL-C (mmol/L)	2.76 (2.34–3.315)	2.75 (2.245–3.415)	0.570
Uric acid (umol/L)	324 (269.5–381.5)	300 (254–344)	0.002
Homocysteine (umol/L)	12.57 (10.4–16.3)	12.8 (10.53–16.565)	0.317
Memory (score)	17 (13–22)	12 (4–14)	<0.001
Language (score)	43 (38–51)	34 (25–38)	<0.001
Execution (score)	171 (139–213)	216 (204–294)	<0.001
Visuospatial (score)	4 (3–4)	2 (1–3)	<0.001

Data are expressed as median (quartile 25, quartile 75) or number (proportion).

PSCI, post-stroke cognitive impairment; PSNCI, post-stroke no cognitive impairment; TOAST, trial of org 10172 in acute stroke treatment; LAA, large artery atherosclerosis; CE, cardioembolism; SVO, small vessel occlusion; OD, other determined; UD, undetermined; NIHSS, National Institute of Health Stroke Scale; DMTS, diameter of maximum transverse section; PVH, periventricular hyperintensity; DWMH, deep white matter hyperintensity; HDL-C, high-density lipoprotein cholesterol; LDL-C, low-density lipoprotein cholesterol.

**Figure 2:**
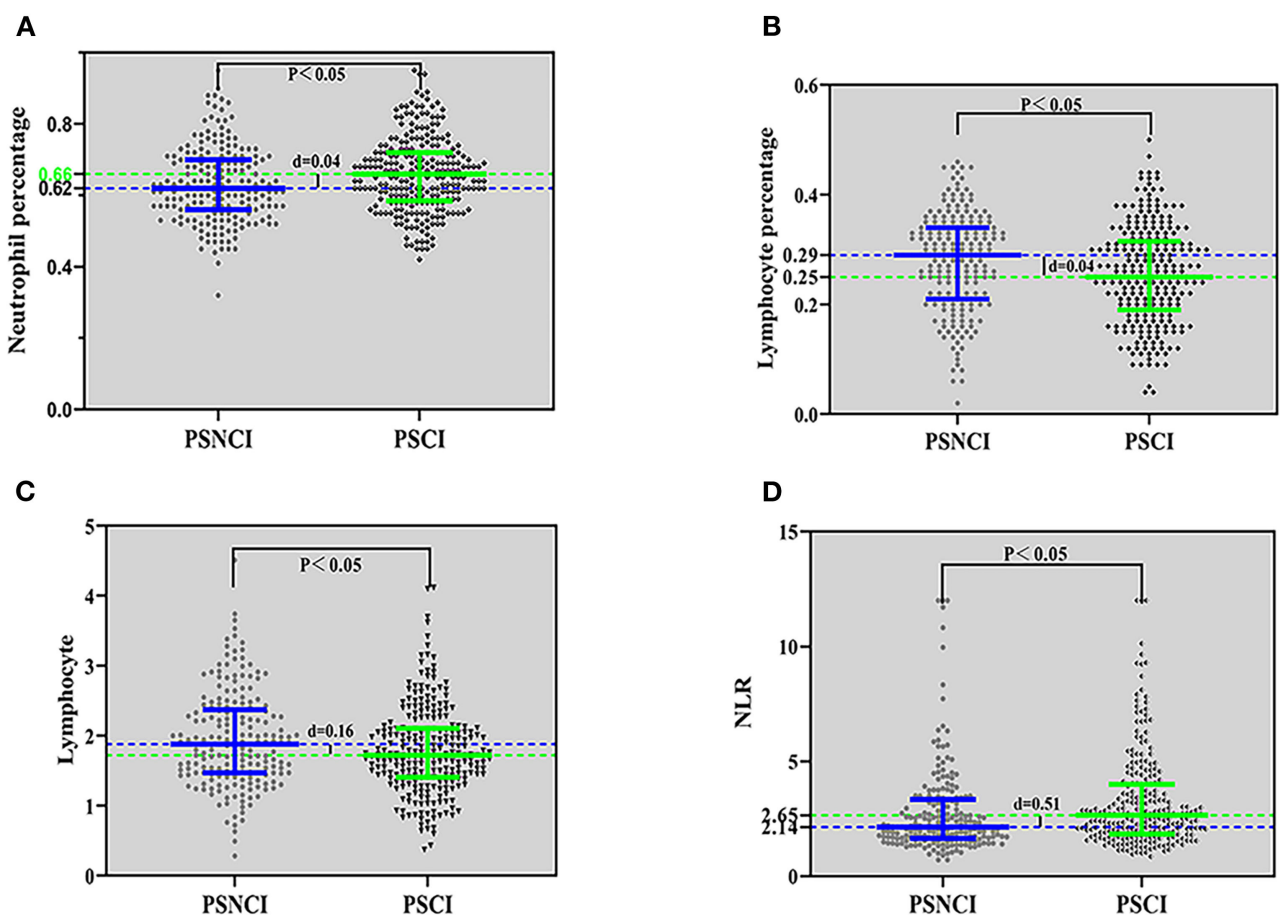
Comparison of inflammatory indicators between the PSCI and PSNCI groups, based on the neutrophil percentage **(A)**, lymphocyte percentage **(B)**, lymphocyte **(C)**, and NLR **(D)**. PSCI, post-stroke cognitive impairment; PSNCI, post-stroke no cognitive impairment; NLR, neutrophil/lymphocyte ratio.

A comparison between the PSCI and PSNCI groups regarding peripheral blood inflammatory markers (see Figure 2) revealed that the percentage of neutrophils and lymphocytes, absolute value of lymphocytes, and NLR were statistically different (*P* < 0.05); the percentage of neutrophils (0.66) and NLR (2.65) in the PSCI group was higher than that in the PSNCI group (0.62, 2.14), and the other two indicators were lower than that in the PSNCI group.

Considering that NLR, a continuous numerical variable, was statistically different between the two groups of PSCI and PSNCI, we further assessed whether there were differences across the two groups at different NLR levels. As represented in Table 2, the incidence of PSCI gradually increased with a higher NLR, and the trend chi-square test suggested that the difference between different NLR classes was statistically significant (*P* < 0.05).

**Table 2:** Comparison of baseline characteristics between different groups in NLR quintiles.

	**Q1 (≤1.62)**	**Q2 (1.63–2.08)**	**Q3 (2.09–2.72)**	**Q4 (2.73–4.04)**	**Q5 (≥4.05)**	***P*-value**
	***N* = 89**	***N* = 91**	***N* = 94**	***N* = 89**	***N* = 91**	
PSCI (*N*, %)	42 (47.2)	43 (47.3)	52 (55.3)	56 (62.9)	60 (65.9)	**0.027**
Sex-Female (*N*, %)	28 (31.5)	27 (29.7)	29 (30.9)	22 (24.7)	22 (24.2)	0.708
Age (years)	61 (57–65.5)	61 (56–67)	62 (55–66)	63 (57–70)	64 (58–70)	0.072
Education (years)	12 (9–12)	9 (6–12)	9 (9–12)	9 (9–12)	9 (6–12)	0.870
Hypertension (*N*, %)	52 (58.4)	54 (59.3)	55 (58.5)	66 (74.2)	63 (69.2)	0.080
History of stroke (*N*, %)	26 (29.2)	22 (24.2)	26 (27.7)	23 (25.8)	23 (25.3)	0.946
Stroke subtype (TOAST)						0.194
LAA (*N*, %)	45 (56.0)	45 (49.5)	43 (45.7)	51 (57.3)	53 (58.2)	
CE (*N*, %)	3 (3.4)	0 (0.0)	1 (1.1)	1 (1.1)	0 (0.0)	
SVO (*N*, %)	36 (40.4)	39 (42.9)	40 (42.6)	32 (36.0)	24 (26.4)	
OD (*N*, %)	1 (1.1)	2 (2.2)	1 (1.1)	1 (1.1)	3 (3.3)	
UD (*N*, %)	4 (4.5)	5 (5.5)	9 (9.6)	4 (4.5)	11 (12.1)	
Number of infarcts (≥ 2) (*N*, %)	43 (48.3)	39 (42.9)	44 (46.8)	37 (41.6)	41 (45.1)	0.893
Anterior circulation infarction (*N*, %)	50 (56.2)	51 (56.0)	47 (50.0)	40 (44.9)	37 (40.7)	0.264
Systolic pressure (mmHg)	145 (135–161)	142 (131–164)	150 (138–167)	150 (137–166)	152 (137–167)	0.113
DMTS (cm)	12.9 (9.2–14.1)	11.3 (7.6–14.1)	12.7 (8.9–16.1)	11.4 (7.3–14.1)	14.0 (10.0–20.1)	**0.007**
NIHSS (score)	2 (1–3)	2 (1–3)	2 (1–3)	2 (1–4)	2 (1–3)	0.621
DWMH	1 (1–1)	1 (1–1)	1 (0–1)	1 (1–2)	1 (1–2)	**0.022**
Uric acid (umol/L)	321 (270–367)	308 (264–366)	298 (249–360)	314 (268–366)	309 (268–359)	0.264
Memory (score)	14 (11–18)	13 (9–18)	13 (9–18)	13 (8–19)	12 (4–18)	0.131
Language (score)	38 (34–48)	38 (30–44)	38 (32–45)	38 (30–44)	37 (29–42)	0.161
Execution (score)	202 (159–229)	204 (155–238)	213 (160–246)	213 (171–256)	213 (179–269)	**0.038**
Visuospatial (score)	3 (2–4)	3 (2–4)	3 (1–4)	3 (1–4)	2 (1–4)	0.088

Data are expressed as median (quartile 25, quartile 75) or number (proportion).

Q1, first NLR quintile; Q2, second NLR quintile; Q3, third NLR quintile; Q4, fourth NLR quintile; Q5, fifth NLR quintile; N, number of patients; PSCI, post-stroke cognitive impairment; TOAST, trial of org 10172 in acute stroke treatment; LAA, large artery atherosclerosis; CE, cardioembolism; SVO, small vessel occlusion; OD, other determined; UD, undetermined; NIHSS, National Institute of Health Stroke Scale; DMTS, diameter of maximum transverse section; DWMH, deep white matter hyperintensity; HDL-C, high-density lipoprotein cholesterol; LDL-C, low-density lipoprotein cholesterol. Bold represent 0.027, <0.05; 0.007, <0.05; 0.022, <0.05; 0.038, <0.05.

In summary, the peripheral blood inflammatory index, neutrophil percentage, NLR, and NLR quintile were statistically different between the two groups of PSCI and PSNCI (*P* < 0.05).

### Analysis of independent factors influencing PSCI in the acute phase of AIS

**Figure 3:**
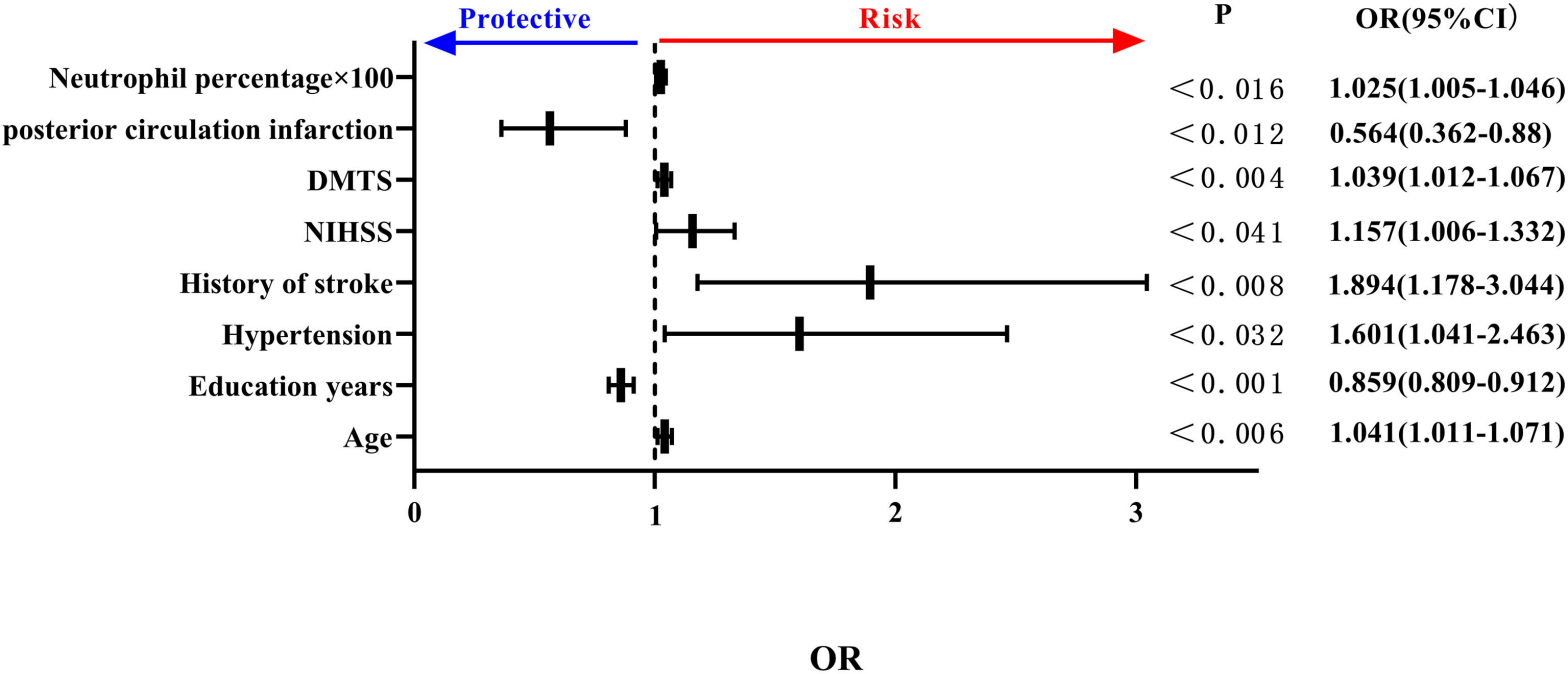
Forest plot of PSCI impact factors (incorporating percentage of neutrophils). PSCI, post-stroke cognitive impairment.

Considering the possible covariance between peripheral blood inflammatory indicators, separate binary logistic regression analyses were carried out for the different inflammatory indicators. The percentage of neutrophils and lymphocytes included in peripheral blood inflammatory indicators, such as age, anterior circulation infarction, previous history of hypertension, history of stroke, NIHSS score, DMTS, and neutrophil percentage, were all independent risk factors for the development of PSCI, and high educational level was an independent protective factor for PSCI. Specifically, each 0.01 increase in neutrophil percentage was associated with a 1.025-fold increase in the likelihood of PSCI (see Figure 3).

**Figure 4:**
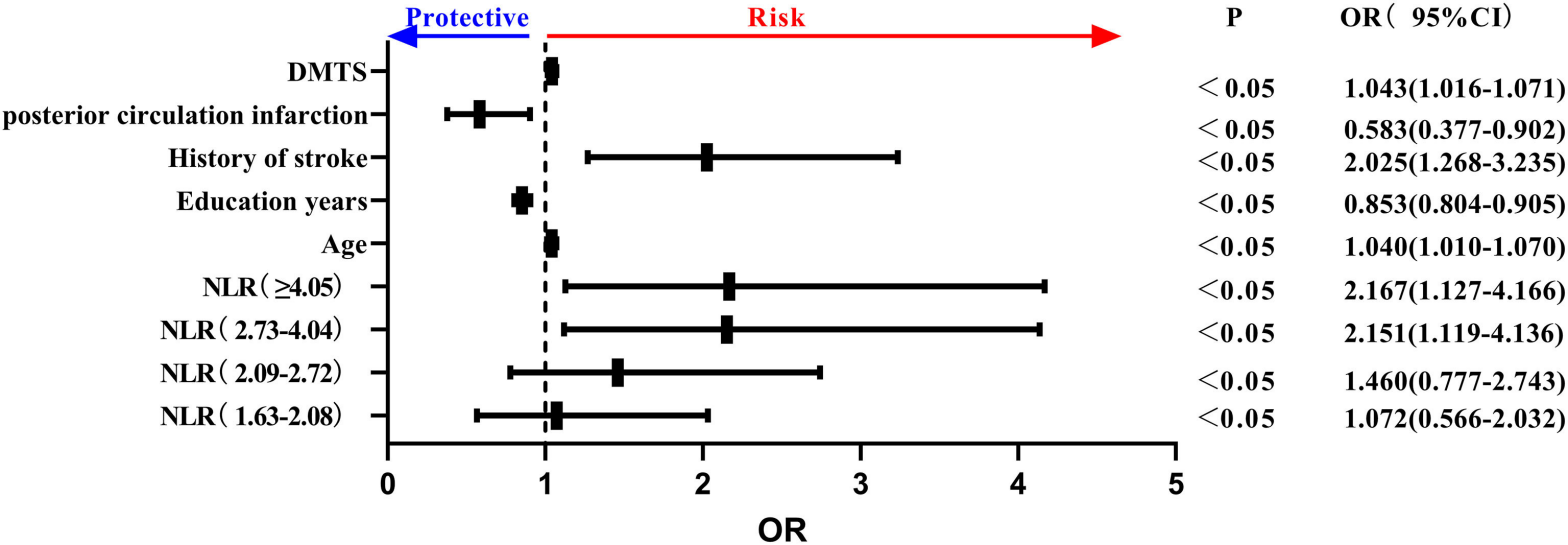
Forest plot of PSCI impact factors (incorporating NLR quintiles). PSCI, post-stroke cognitive impairment; NLR, neutrophil/lymphocyte ratio.

Key NLR quintiles were included among peripheral blood inflammatory markers. Age, history of stroke, DMTS, anterior circulation cerebral infarction, and NLR as a class variable were all independent risk factors for the development of PSCI, and educational level was an independent protective factor for PSCI. Specifically, the risk of PSCI was 2.151 times higher in NLR (2.73–4.04) than in NLR (<1.62), and 2.167 times higher in NLR (≥4.05) than in NLR (≤1.62) (*P* = 0.001 <0.05) and in the trend chi-square test. The risk of PSCI increased as NLR increased (see Figure 4).

In summary, the neutrophil percentage and NLR quintile represented independent risk factors for PSCI. Furthermore, we found that four indicators, namely, age, education level, previous stroke history, and DMTS, were independent influencing factors associated with PSCI.

### Predictive value of various inflammatory indicators of peripheral blood for PSCI in the acute phase of AIS

**Figure 5:**
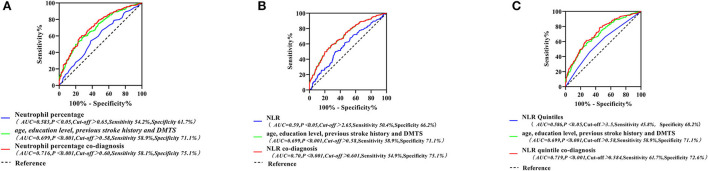
Neutrophil percentage and its combination with other indicators for the ROC curve of PSCI **(A)**; ROC curves of NLR and its combined other indicators for PSCI **(B)**; ROC curves for the NLR quintile and its combination with other indicators for the PSCI **(C)**. PSCI, post-stroke cognitive impairment; NLR, neutrophil/lymphocyte ratio; ROC, receiver operating characteristic curve.

Using ROC curves to assess the diagnostic value of neutrophil percentage, NLR, and NLR quintile for PSCI, we identified that there were limitations in the sensitivity and specificity of a single peripheral blood inflammatory marker for predicting PSCI. Specifically, the neutrophil percentage had a diagnostic accuracy, sensitivity, and specificity of 58.3, 54.2, and 61.7%, respectively, while those of NLR were 59, 50.4, and 66.2%, respectively, and those of the NLR quintile were 58.6, 45.8, and 68.2% for PSCI. Four indicators, namely, age, education level, previous stroke history, and DMTS, were also found to be independent influencing factors for PSCI. The diagnostic accuracy, sensitivity, and specificity of PSCI were 69.9, 58.9, and 71.1%, respectively. Therefore, we combined each inflammatory marker with the above four indicators to build new models and probe the diagnostic value of each new model for PSCI. The accuracy, sensitivity, and specificity of neutrophil percentage co-diagnoses were 71.6, 58.1, 75.1, respectively; those for NLR co-diagnoses were 70.0, 54.9, and 75.1%, respectively; and those for the NLR quintile co-diagnoses were 71.9, 61.7, and 72.6%, respectively. The results revealed that the area under the curve, sensitivity, and specificity of the new models, including inflammatory indicators, were significantly higher for predicting PSCI than for the single inflammatory markers (as presented in Figure 5).

## Discussion

At present, there are few studies on the normal values of NLR in a healthy population of which, one demonstrates (14) that normal NLR values in adults in the healthy non-elderly population are between 0.78 and 3.53. The study population was aged mainly 50–80 years with a median NLR of 2.39. Based on our findings, the percentage of neutrophils (0.66) and NLR (2.65) were higher in the PSCI group than in the PSNCI group (0.62, 2.14). In addition, the neutrophil ratio and NLR quintile were independent risk factors for PSCI. After controlling for other factors, each 0.01 increase in neutrophil percentage was associated with a 1.025-fold increase in the likelihood of PSCI. Compared with that in the lowest quintile of NLR, Q1 (≤1.62), the risk of PSCI was 2.151 times higher in Q4 (2.73–4.04) and 2.167 times higher in Q5 (≥ 4.05); the higher the neutral ratio and NLR, the higher the incidence of PSCI and the more severely impaired executive function. These findings suggest that systemic inflammation may play a key role in the pathogenesis of PSCI in the acute phase of AIS.

A Korean study (15) also found that NLR in the acute phase of stroke was independently associated with an increased risk of PSCI at 3 months and that the highest quintile (NLR ≥ 3.80) increased the risk of PSCI by 3.26-fold compared with that of the lowest quintile ( ≤ 1.57). Other studies, meanwhile, have demonstrated that an elevated NLR is independently associated with an increased risk of AD in people aged > 65 years (16), with a correlation between NLR and CI (13, 17, 18). A Chinese study of the mild cognitive impairment (MCI) population (19) revealed that NLR was significantly higher in the MCI group than in that with normal cognitive function, with subjects having an NLR ≥ 2.07 with a nearly six-fold increased risk relative to those with an NLR < 2.07. Although NLR has different diagnostic thresholds for cognitive disorders, a relatively consistent relationship between NLR and CI has been documented, and systemic inflammation may play a critical role in the pathogenesis of CI.

When AIS occurs, circulating neutrophils are first recruited to areas of ischemia, inducing a destructive cascade response, while stress conditions lead to a decrease in lymphocyte numbers. Therefore, as a combination of these two different inflammatory markers, the peripheral blood NLR can synthetically represent the systemic inflammatory state of AIS. Few studies have investigated the correlation between neutrophil percentage, NLR and PSCI. Combining the existing studies on neutrophils and lymphocytes in AIS, we suggest that inflammatory substances (ROS, MPO, NE, etc.) released by neutrophils at different stages of AIS may be involved in the development of PSCI by causing hypoperfusion, BBB destruction, brain parenchymal damage, and reduced neovascularization, affecting the structural integrity of the neurovascular unit (5, 6, 20–23). Meanwhile, secondary brain injury resulting from reduced lymphocyte defense against the host may exacerbate neuronal damage and trigger cognitive dysfunction during the recovery phase (15). Although the underlying mechanisms between NLR and cognition remain unclear, systemic immune system overreaction plays an important role in PSCI (15), and peripheral blood NLR is a potentially viable tool for assessing cognitive impairment after stroke. Another important mechanism may be that memory-related regions, such as the hippocampus and parahippocampal gyrus, are more susceptible to ischemic damage and inflammatory disruption in a sustained proinflammatory context. Learning and memory functions in the hippocampus are closely related to the degree of neurogenesis, and animal-based experiments have shown that neuroinflammation inhibits neurogenesis, whereas NSAIDs can restore neurogenesis and thus improve memory by blocking endotoxin-induced inflammation (24). Another study on brain morphology found that inflammation was negatively correlated with the volume of the hippocampus, pallidum, and thalamus and that higher peripheral inflammation (IL-6 and CRP) was associated with poorer spatial reasoning, short-term memory, language skills, learning and memory, and executive function (25). ESR is associated with reduced hippocampal volume and cognitive decline following a stroke (26). The above studies on the association between inflammation and PSCI may be based on central inflammation, with peripheral inflammation as the basis. Peripheral inflammatory mediators such as IL-6 can cross the BBB, leading to neurodegeneration and central inflammatory processes that impair cognitive function (24). Central inflammation interferes with neurogenesis, synaptic plasticity, and neurotransmission, which in turn leads to brain atrophy and cognitive impairment (27). Consequently, higher peripheral blood neutrophil percentage and NLR levels in patients with acute AIS may correspond with a greater central inflammatory response and greater ischemia and atrophy in areas such as the hippocampus associated with memory, which in turn causes cognitive decline in the acute and recovery periods. In addition, it has been suggested that NLR is associated with atherosclerosis, that proinflammatory-activating molecules released by neutrophils in the vascular wall may promote atherosclerosis by reducing nitric oxide (NO), and that B cell-mediated immune regulation may prevent atherosclerosis. Thus, a high NLR resulting from high neutrophil and low lymphocyte counts in inflammatory states may promote atherosclerosis, leading to cognitive decline due to cerebral small vessel disease such as microinfarction (28, 29).

The NLR as a marker of systemic inflammation is more responsive to clinical significance than neutrophils and lymphocytes alone, and it is useful (30), low cost, and widely used, thereby having excellent clinical potential. We assessed the diagnostic value of neutrophil percentage and NLR quintile for PSCI by ROC curves separately and found that the diagnostic accuracy was 58.3–59.0%; at the same time, the sensitivity and specificity were fair at 45.8–68.2%. This indicates that the peripheral blood neutrophilic ratio and NLR in the acute phase of AIS have predictive value for PSCI. This study also found that age, education, history of previous stroke, and DMTS each represented independent factors influencing PSCI. For each 1-year increase in age, the risk of developing PSCI was 1.038–1.046 times higher. One study (31) proposed that the prevalence of PSCI increases exponentially with age after 65 years. A low educational level increases the risk of developing PSCI. However, for each year's increase in educational level, the likelihood of PSCI is 84.9–85.9%. Other studies (32) also suggest that a higher education level may be associated with a better cognitive performance by providing cognitive reserve and increasing tolerance for cognitive decline. Consequently, a new model was developed by combining neutrophil percentage and NLR quintile with all these four indicators. It was found that the new model achieved a diagnostic accuracy of >70% for each model compared to that with the single inflammatory indicator; moreover, the sensitivity and specificity were significantly higher.

We demonstrated that peripheral blood inflammatory indicators in the acute phase of AIS can be used as predictors of the development of PSCI and have diagnostic value, providing a new idea for clinical research related to peripheral immunity and PSCI. Among them, the cognitive assessment in the acute phase of stroke was conducted on days 7–10 after stroke, and during this period, the symptoms of cerebral infarction are relatively stable, which can avoid the interference of cognitive stress damage caused by stroke itself. However, this study has some limitations. First, our data were acquired from a single center. Second, as PSCI could happen right after the stroke and last for 6 months, the follow-up evaluation of the CI is critical, other than 7–10 days post stroke. We also performed cognitive follow-up of some patients at 3 months, 6 months, and 1 year after stroke. Due to the global outbreak of COVID-19 during this period, most patients could not come to the hospital on time to complete the follow-up on time. Therefore, our cognitive follow-up data were insufficient and inconsistent. In future studies, we will refine the analysis at 3 and 6 months after stroke to get a better understanding of the association of peripheral immunization with PSCI. Afterward, we did not collect specific cognitive level and laboratory indicators before stroke. However, we asked about a detailed medical history of the study population, excluding people who were diagnosed with dementia before stroke or who had used drugs affecting cognitive impairment in the 2 weeks before screening, excluding related diseases affecting peripheral blood routine. We hope to design and collect data of patients before stroke in future studies to obtain the higher-quality clinical research result. Finally, the diagnostic accuracy of the peripheral blood index in the acute stroke phase for PSCI was moderate, and further research is expected to explore its real value. Screening patients at high risk of cognitive impairment using acute stroke inflammatory markers combined with history and imaging may enable a future follow-up to be completed 3–6 months following a stroke, with data collected on routine blood and cognitive assessments, to establish a longitudinal cohort study to dynamically observe the potential value of inflammatory markers for the diagnosis of PSCI.

In conclusion, neutrophil percentage and NLR in the acute phase of AIS were independently associated with PSCI. In addition, a high NLR was strongly associated with executive function. In conclusion, neutrophil percentage and NLR provide a certain diagnostic value for PSCI, which is of key clinical relevance.

## Data availability statement

The original contributions presented in the study are included in the article/supplementary material, further inquiries can be directed to the corresponding author.

## Ethics statement

The studies involving human participants were reviewed and approved by The First Hospital of Jilin University. The patients/participants provided their written informed consent to participate in this study.

## Author contributions

TS analyzed the literature and wrote the manuscript. YS, CW, ZW, WZ, ML, and YW collected the data and designed the study. BM contributed to manuscript editing. LS provided the framework for the study and reviewed and edited the manuscript. All authors have read and agreed to the final version of the manuscript.

## Funding

This study was funded by the Ministry of Science and Technology's major chronic disease project of China (No. 2018YFC1312301).

## Conflict of interest

The authors declare that the research was conducted in the absence of any commercial or financial relationships that could be construed as a potential conflict of interest.

## Publisher's note

All claims expressed in this article are solely those of the authors and do not necessarily represent those of their affiliated organizations, or those of the publisher, the editors and the reviewers. Any product that may be evaluated in this article, or claim that may be made by its manufacturer, is not guaranteed or endorsed by the publisher.
